# Combined use of the entomopathogenic fungus, *Metarhizium brunneum*, and the mosquito predator, *Toxorhynchites brevipalpis,* for control of mosquito larvae: Is this a risky biocontrol strategy?

**DOI:** 10.1016/j.jip.2018.02.003

**Published:** 2018-03

**Authors:** Abeer M. Alkhaibari, Thierry Maffeis, James C. Bull, Tariq M. Butt

**Affiliations:** a Department of Biosciences, College of Science, Swansea University, Singleton Park, Swansea, United Kingdom; b Centre for Nanohealth, College of Engineering, Swansea University, Swansea, United Kingdom; c Department of Biology, Faculty of Science, Tabuk University, Saudi Arabia

**Keywords:** *Aedes*, *Metarhizium*, *Toxorhynchites*, Predator, Fungal pathogen, Blastospores, Conidia, Risk assessment, Interaction

## Abstract

Mosquitoes transmit several diseases, which are of global significance (malaria, dengue, yellow fever, Zika). The geographic range of mosquitoes is increasing due to climate change, tourism and trade. Both conidial and blastospore formulations of the entomopathogenic fungus, *Metarhizium brunneum* ARSEF 4556, are being investigated as mosquito larvicides. However, concerns have been raised over possible non-target impacts to arthropod mosquito predators such as larvae of *Toxorhynchites brevipalpis* which feed on larvae of mosquito vector species. Laboratory-based, small container bioassays showed, that *T. bevipalpis* larvae are susceptible to relatively high concentrations (i.e. ≥10^7^ spores ml^−1^) of inoculum with blastospores being significantly more virulent than conidia. At lower concentrations (e.g. <10^7^ spores ml^−1^), it appears that *M. brunneum* complements *T. brevipalpis* resulting in higher control than if either agent was used alone. At a concentration of 10^5^ spores ml^−1^, the LT_50_ of for conidia and blastospores alone was 5.64 days (95% CI: 4.79–6.49 days) and 3.89 days (95% CI: 3.53–4.25 days), respectively. In combination with *T. brevipalpis*, this was reduced to 3.15 days (95% CI: 2.82–3.48 days) and 2.82 days (95% CI: 2.55–3.08 days). Here, combined treatment with the fungus and predator was beneficial but weaker than additive. At 10^7^ and 10^8^ blastospores ml^−1^, mosquito larval mortality was mostly due to the fungal pathogen when the predator was combined with blastospores. However, with conidia, the effects of combined treatment were additive/synergistic at these high concentrations. Optimisation of fungal concentration and formulation will reduce: (1) risk to the predator and (2) application rates and costs of *M. brunneum* for control of mosquito larvae.

## Highlights

• *Metarhizium brunneum* is highly pathogenic to *Aedes aegypti* larvae.
• *Metarhizium* blastospores more virulent than conidia.
• Mosquito predator, *Toxorhynchites brevipalpis*, is more tolerant than *Aedes* to *Metarhizium.*
• *Metarhizium* and *Toxorhynchite*s combination gives excellent control of *Aedes* larvae.
• *Metarhizium* risk to predator is reduced when inoculum is used at low concentrations.

## Graphical abstract

**Figure:**
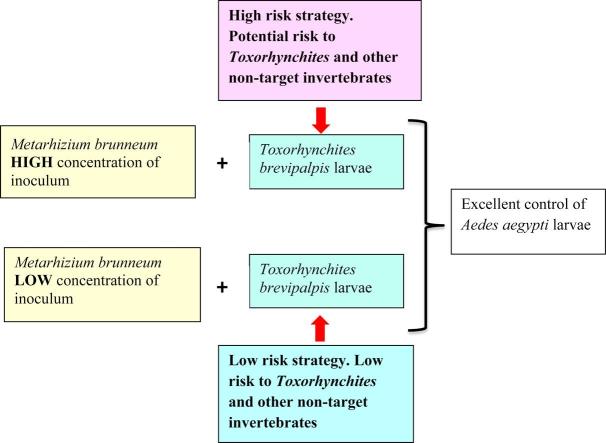

## 1 Introduction

Mosquitoes belonging to the genera *Aedes*, *Anopheles* and *Culex* vector a range of diseases (e.g. malaria, Zika, dengue, yellow fever), which have significant medical and economic impacts for over half the world’s population (Tolle, 2009). *Aedes* mosquitoes will oviposit in extremely small, ephemeral bodies of water since their eggs can tolerate desiccation (Faull et al., 2016, Juliano et al., 2002). Current control methods targeting adult mosquitoes include persistent insecticide-treated nets and indoor residual spraying. However, targeting adults alone is insufficient in preventing disease transmission, and integrated vector management (IVM) focuses on management of both larval and adult mosquito populations (Fillinger et al., 2009, Thomas, 2017). Various tools are available to control mosquito larvae in large expanses of water such as larvivorous fish and chemical pesticides including growth regulators such as methoprene (Becker et al., 2003). More selective insecticides based on the bacteria *Bacillus sphaericus* and *Bacillus thuringiensis israelensis* are also widely used especially in urban and environmentally sensitive areas (Lacey, 2007, Mulla, 1990). However, when dealing with transient or small bodies of water (e.g. water collected at the bottom of used tyres or in leaf clusters of epiphytic plants such as bromeliads) the products and strategies are more limited (Ceretti-Junior et al., 2016).

There is a reluctance to use chemical insecticides, even though they are relatively fast acting, because of the risks they pose to human health and pollution of the environment even at relatively low concentrations (Liess et al., 2013). Furthermore, extensive use of agricultural chemical pesticides can select for insecticide resistance in mosquito disease vectors (Nkya et al., 2014). Indeed, use of both chemical and bacterial insecticides is under threat due to increasing reports of mosquitoes developing resistance to these agents (Boyer et al., 2012, Hemingway and Ranson, 2000). These factors are prompting the search for safe alternatives such as the entomopathogenic fungi (EPF) (Shah and Pell, 2003). Laboratory studies show that *Metarhizium brunneum* can cause up to 100% mortality of mosquito larvae in <24 h depending on the fungal strain, formulation and concentration (Alkhaibari et al., 2017, Greenfield et al., 2015). However, there are many other EPF species which have been shown to infect mosquito eggs, larvae and adults including species of *Tolypocladium cylindrosporum*, *Beauveria bassiana* and *Metarhizium ansiopliae* (Scholte et al., 2004).

Conidia and blastospores of *M. brunneum* differ in their mode of pathogenesis (Alkhaibari et al., 2016, Butt et al., 2013). Conidia are unable to infect through the cuticle due to their failure to adhere to the surface of the mosquito larval cuticle (Greenfield et al., 2014). However, conidia are readily ingested and although they do not germinate in the gut lumen, they can cause death through stress-induced apoptosis triggered by the spore bound protease Pr1 (Butt et al., 2013). In contrast, blastospores readily adhere to the host cuticle and are also ingested. These propagules quickly germinate with death resulting from simultaneous penetration of the cuticle and gut and subsequent colonisation of the haemocoel (Alkhaibari et al., 2016).

The use of EPF offers reduced risk to aquatic systems compared with many alternatives, for example through reduced “run off” from forest slopes or agricultural land (Ippolito et al., 2015). However, some concerns over non-target impacts of EPF have been raised. Toxicology studies show that the risk posed by *M. brunneum* conidia to the aquatic invertebrates *Artemia salina* and *Daphnia pulex* is concentration-dependent, that is, mortality increased with spore concentration (Garrido-Jurado et al., 2015). Since these invertebrates were far more tolerant of *M. brunneum* than mosquito larvae it was possible to identify a concentration which gave effective control of the pest with significantly reduced risk to the non-target invertebrates (Garrido-Jurado et al., 2015). No study has been conducted to date to determine the risk posed by EPF to the aquatic invertebrate predatory mosquito *Toxorhynchites* even though this genus is widely recognised as an important biological control agent (BCA) (Shaalan and Canyon, 2009). In fact, there are no studies on the combined use of EPF and predacious insects for mosquito control even though the potential exists to enhance mosquito control using combinations. In contrast, there are several studies on the combined use of EPF and other BCAs for control of agricultural pests (Dogan et al., 2017). The combined used of EPF with these BCAs is increasingly being used within integrated pest management (IPM) programmes partly because these agents may act in concert, allowing each agent to be used at reduced application rates. For example, co-application of *M. brunneum* with EPN resulted in higher mortality of black vine weevil (*Otiorhynchus sulcatus*) larvae than if either agent was used alone (Ansari et al., 2008). Similarly, other researchers have reported pest control being enhanced when using EPF-predator combinations whether targeting foliar or subterranean pests (Roy and Pell, 2000, Saito and Brownbridge, 2016). Most often the success of these combinations has been attributed to predators either avoiding the pathogen or being less susceptible to it compared with the target pest (Dogan et al., 2017, Meyling and Pell, 2006, Ormond et al., 2011). Successful IPM programmes aim to exploit compatible, synergistic combinations of EPF and beneficial predators to reduce application rates and costs and concomitantly reduce risks to non-target organisms.

Species of the predatory mosquito, *Toxorhynchites,* are found in diverse habitats feeding on vector prey species (Collins and Blackwell, 2000). *Toxorhynchites* species are efficient predators and can eliminate mosquito larvae where present (Shaalan and Canyon, 2009). However, to date, no studies have investigated the compatibility of *Toxorhynchites* with EPF. The aims of this study were to: (1) determine the susceptibility of *Toxorhynchites brevipalpis* to *Metarhizium brunneum* ARSEF 4556 and (2) establish if *M. brunneum* and *T. brevipalpis* could work together through manipulation of the fungal inoculum concentration and formulation. The significance of this study to the development of IVM programmes is discussed.

## 2 Materials and methods

### 2.1 Maintenance of *Aedes aegypti* and *Toxorhynchites brevipalpis*

Eggs of both *Aedes aegypti* (AEAE) and *Toxorhynchites brevipalpis* (TOXO) were obtained from the London School of Hygiene and Tropical Medicine and hatched in 1L and 3L tap water, respectively. Larvae of *A. aegypti* were fed guinea pig pellets (PetsAtHome, Swansea, UK). Larvae of *T. brevipalpis* were isolated in 100 ml water within 2–3 days to avoid cannibalism and provided 5 *A. aegypti* larvae daily as food. Throughout the study, *T. brevipalpis* were fed with *A. aegypti* larvae of the same instar as the predator (Mohamad and Zuharah, 2014). The insects were maintained at 27 ± 1 °C with 12L: 12D photoperiod. Fourth instar *T. brevipalpis* and *A. aegypti* were used in the assays outlined below.

### 2.2 Conidia and blastospore production

Conidia of *M. brunneum* ARSEF 4556 and a green fluorescence protein (GFP) transformed strain of *M. brunneum* EAMa 01/58 Su were harvested from 14 day old cultures produced on Sabouraud Dextrose Agar (SDA). Strain ARSEF 4556 was obtained from the USDA-ARS culture collection while EAMa 01/58 Su was provided by Prof Quesada-Moraga, University of Cordoba, Spain. Blastospores were produced in Adamek’s medium as outlined by Alkhaibari et al. (2016). Conidia and blastospores concentrations were determined using an improved Neubauer haemocytometer and diluted to the desired concentration using 0.03% Aq Tween and distilled water, respectively.

### 2.3 Susceptibility of *T. Brevipalpis* and *A. Aegypti* larvae to *M. Brunneum*

The susceptibility of *T. brevipalpis* larvae to conidia and blastospores suspensions of *M. brunneum* ARSEF 4556 was tested in 200 ml plastic cups containing 100 ml of water with 30 larvae per treatment i.e. per concentration. Conidia and blastospores were suspended in 0.03% Aqueous Tween 80 and distilled water, respectively, before applying to the bioassay cups for a final concentration of 10^5^, 10^6^, 10^7^ spore ml^−1^. Each larva of *T. brevipalpis* was provided ten *A. aegypti* larvae at the start of each assay. Controls consisted of either distilled water or Tween 80 at final concentration 0.0003% (v/v). Mortality was recorded daily over7 days. A total of 240 *T. brevipalpis* larvae were used across all experiments.

Assays were also conducted to determine *A. aegypti* susceptibility to both conidia and blastospores of *M. brunneum* as described by Alkhaibari et al. (2017). Briefly, three replicates of ten larvae (n = 30) per treatment were transferred to plastic cups containing 100 ml of conidia or blastospores suspension at final concentrations of 10^5^, 10^6^, 10^7^ spores ml^−1^. Mortality was assessed daily for 7 days. In total, 420 *A. aegypti* larvae were used in this study. Each experiment was repeated three times.

### 2.4 Microscopy studies

The infection and developmental processes of *M. brunneum* in *T. brevipalpis* larvae was investigated using a combination of low-temperature scanning electron microscopy (Cryo-SEM) and fluorescence microscopy. For Cryo-SEM, larvae were inoculated with conidia and blastospores of *M. brunneum* ARSEF 4556 as described above (at concentration 10^7^ spores ml^−1^ for 24 h) then examined using a Hitachi S4800 field emission microscope equipped with a Quorum PPT2000 cryogenic stage and preparation chamber, as outlined by Alkhaibari et al. (2016). For fluorescence microscopy, *T. brevipalpis* larvae (n = 5) were fed *Aedes* larvae infected with conidia and blastospores of a GFP-transformed strain of *M. brunneum* (10^7^ spores ml^−1^). This facilitated visualisation of the inoculum in the digestive tract and faecal pellets and concomitantly allowed the viability of inoculum to be determined. The surface and gut contents of infected *A. aegypti* larvae as well as faecal pellets were examined using a Zeiss fluorescence microscope, as outlined by Butt et al. (2013).

### 2.5 Interactions between *M. Brunneum* and *T. Brevipalpis* in control of *A. Aegypti* larvae

Interactions between the predator and fungal pathogen were investigated using different concentrations and formulations of the fungus. Briefly, concentration mortality studies were performed as outlined above using four different concentrations (10^5^, 10^6^, 10^7^, 10^8^ spores ml^−1^) of conidia and blastospores in absence of the predator *T. brevipalpis*. An additional study was conducted using the above concentrations of conidia and blastospores with only a single larva of *T. brevipalpis* being added to each treatment. Control insects were exposed to carrier (distilled water or 0.3% Aq Tween) only. Mortality was recorded daily for 5 days. In total, 600 *A. aegypti* larvae and 30 *T. Brevipalpis* larvae were used in this study. The experiments were repeated three times.

### 2.6 Statistical analyses

Survival rates of (1) *T. brevipalpis* and *A. aegypti* larvae exposed to the different concentrations of *M. brunneum* ARSEF 4556 conidia and blastospores and (2) *A. aegypti* larvae exposed to four concentrations of fungal spores (blastospores and conidia) in presence and absence of *T. brevipalpis* were visualised by plotting Kaplan-Meier survival cumulative survival functions by treatment, with pairwise comparisons assessed using log-rank tests (Kaplan and Meier, 1958) The median lethal time to death, LT_50_, was estimated using parametric survival regression for combinations of fungal formulation, spore concentration, and mosquito species (Crawley, 2012). For the bioassays of the interactions between the fungus and the predator to control *A. aegypti* larvae, the LT_50_ values of the latter were also calculated using parametric survival regression for combinations of fungal formulation, spore concentration, predator (presence/absence). By comparing observed survival following combined treatment with expected survival, based on the additive effects of the fungus and predator alone, we tested whether combined treatment was (a) antagonistic (higher *A. aegypti* survival than expectation), (b) additive, or (c) synergistic (lower *A. aegypti* survival than expectation).

All statistical analyses were carried out using SPSS v22.0 (Morgan et al., 2012) and R Version 3.3.1 (R Core Team, 2012).

## 3 Results

### 3.1 Susceptibility of *T. Brevipalpis* and *A. Aegypti* larvae to *M. Brunneum*

**Fig. 1:**
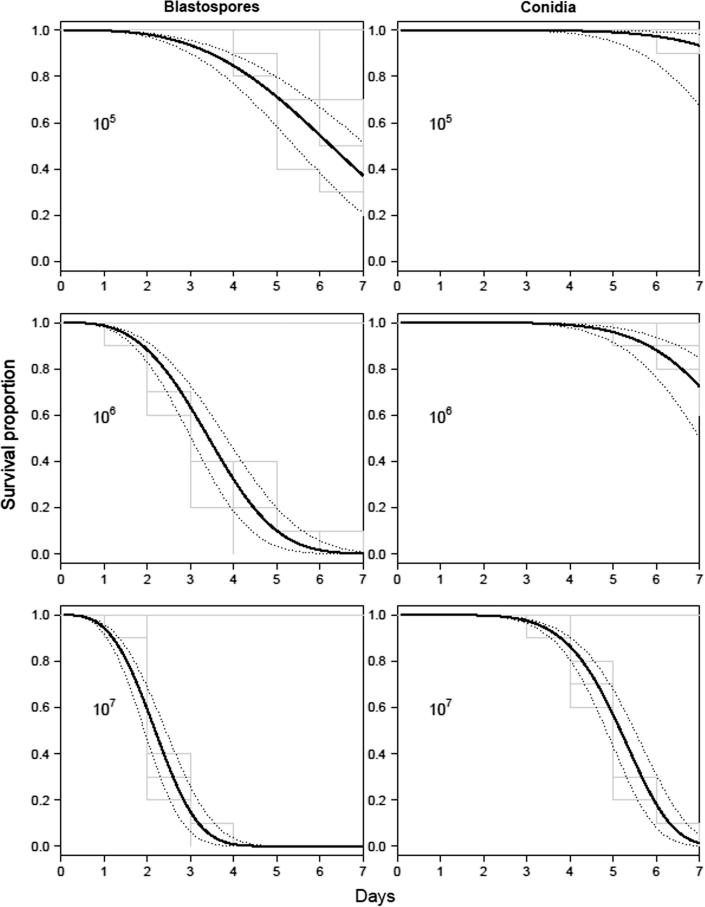
Survival curves of *Toxorhynchites brevipalpis* larvae exposed to different concentrations of conidia and blastospores of *Metarhizium brunneum* ARSEF 4556. Percentage cumulative survival of *Tx. brevipalpis* (L_4_) exposed to different concentrations of *M. brunneum* ARSEF 4556 over a 7 day period. Kaplan–Meier step functions after treatment with 10^5^, 10^6^, or 10^7^ propagules ml^−1^ are shown in gray (including uninfected controls). Fitted survival curves are shown in black, with 95% confidence intervals shown as dotted lines.

**Fig. 2:**
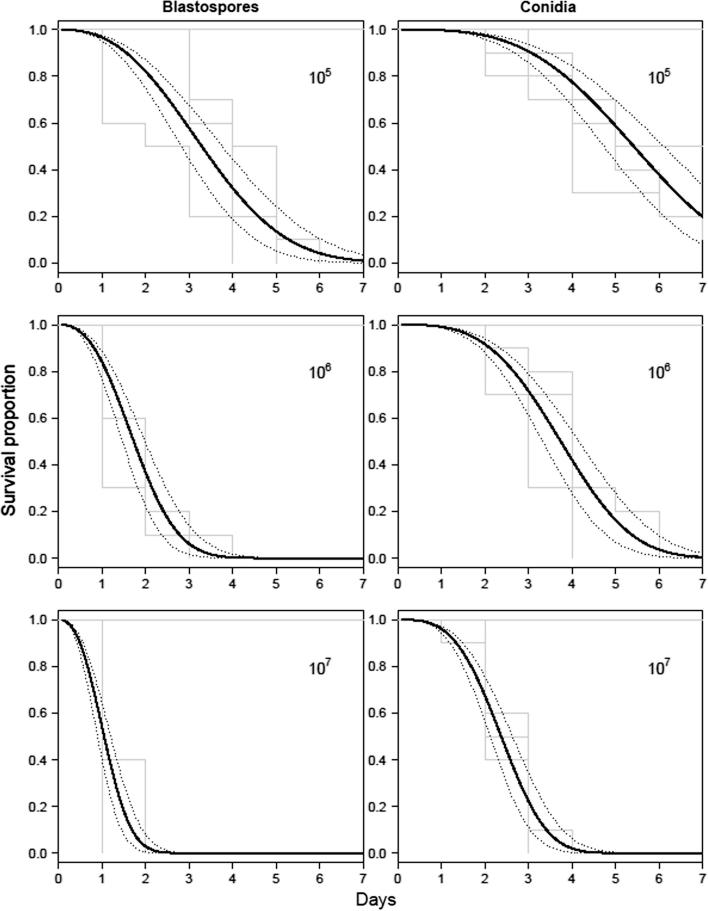
Survival curves of *Aedes aegypti* Larvae exposed to different concentrations of conidia and blastospores of *Metarhizium brunneum* (ARSEF 4556). Percentage cumulative survival of *A. aegypti* exposed to varied concentrations of *M. brunneum* (strain: ARSEF 4556) for 7 days. Kaplan–Meier step functions after treatment with 10^5^, 10^6^, or 10^7^ propagules ml^−1^ are shown in gray (including uninfected controls). Fitted survival curves are shown in black, with 95% confidence intervals shown as dotted line.

Both *T. brevipalpis* and *A. aegypti* were susceptible to *M. brunneum* ARSEF 4556 with mortality being dependent upon the concentration and formulation (Fig. 1, Fig. 2). Larvae of *A. aegypti* were significantly more susceptible to ARSEF4556 compared with *T. brevipalpis*, with the blastospores generally being more virulent than the conidia (Table 1; Fig. 1, Fig. 2). For example LT_50_ values for *A. aegypti* and *T. brevipapis* when exposed to conidia at the highest concentration (10^7^ spore ml^−1^) was 2.7 and 5.5 days, respectively whereas that of blastospores was 1.2 and 2.5 days, respectively (Table 1). *A. aegypti* larvae were generally twice as susceptible to conidia or blastospores than the predator at each concentration tested (Table 1), with pairwise concentration comparisons being statistically significant (Table 2). Both conidia and blastospore applications caused mortalities in both mosquito species significantly higher than the control (*P* < .001). However, for *T. brevipalpis* larvae exposed to conidia at the lowest concentration (10^5^ spores ml^−1^) there was no significant difference with the control (*P* = .154; Table 2; Fig. 1).

**Table 1:** LT_50_ values estimated for *Toxorhynchites brevipalpis* and *Aedes aegypti* larvae versus three concentrations of conidia and blastospores of *Metarhizium brunneum* ARSEF 4556.

Mosquito species	Concentration	Conidia	Blastospores
*Tx. brevipalpis*	1 × 10^5^	10.91 (8.16–13.65)	7.02 (6.08–7.97)
	1 × 10^6^	8.44 (7.45–9.42)	3.85 (3.39–4.30)
	1 × 10^7^	5.50 (5.15–5.84)	2.45 (2.17–2.73)

*A. aegypti*	1 × 10^5^	6.05 (5.29–6.82)	3.81 (3.26–4.35)
	1 × 10^6^	4.18 (3.72–4.64)	2.00 (1.70–2.30)
	1 × 10^7^	2.66 (2.37–2.95)	1.22 (1.04–1.39)

Mean lethal time (LT_50_) for conidia and blastospores against *Tx. brevipalpis* and *A. aegypti* larvae at three concentrations (1 × 10^5^, 1 × 10^6^ and 1 × 10^7^ spore ml^−1^). 95% confidence intervals are given in parenthesis.

**Table 2:** Kaplan-Meier log rank pairwise comparisons of conidia and blastospores concentrations for treatments against *Toxorhynchites brevipalpis* and *Aedes aegypti* larvae.

Mosquito species	Formulations	Conidia			Blastospores		
	Concentrations	10^5^	10^6^	10^7^	10^5^	10^6^	10^7^
*Tx. brevipalpis*	Control	χ^2^ = 2.03 *P* = .154	χ^2^ = 10.40 *P* = .001	χ^2^ = 66.39 *P* < .001	χ^2^ = 32.45 *P* < .001	χ^2^ = 68.19 *P* < .001	χ^2^ = 65.38 *P* < .001
	10^5^	–	χ^2^ = 5.27 *P* = .022	χ^2^ = 61.95 *P* < .001	–	χ^2^ = 38.82 *P* < .001	χ^2^ = 63.63 *P* < .001
	10^6^	–	–	χ^2^ = 49.63 *P* < .001	–	–	χ^2^ = 10.54 *P* = .001

*A. aegypti*	Control	χ^2^ = 35.69 *P* < .001	χ^2^ = 65.62 *P* < .001	χ^2^ = 61.57 *P* < .001	χ^2^ = 65.73 P < .001	χ^2^ = 69.70 P < .001	χ^2^ = 66.26 P < .001
	10^5^	–	χ^2^ = 10.48 *P* = .001	χ^2^ = 36.45 *P* < .001	–	χ^2^ = 26.70 P < .001	χ^2^ = 47.65 P < .001
	10^6^	–	–	χ^2^ = 22.06 *P* < .001	–	–	χ^2^ = 7.51 P = .006

*Tx. brevipalpis* and *A. aegypti* exposed to different concentrations of conidia and blastospores of *M. brunneum*. χ^2^ = *Chi*-square value.

### 3.2 Microscopy studies of conidia and blastospore interactions in the gut and cuticle surface of *T. Brevipalpis* larvae

**Fig. 3:**
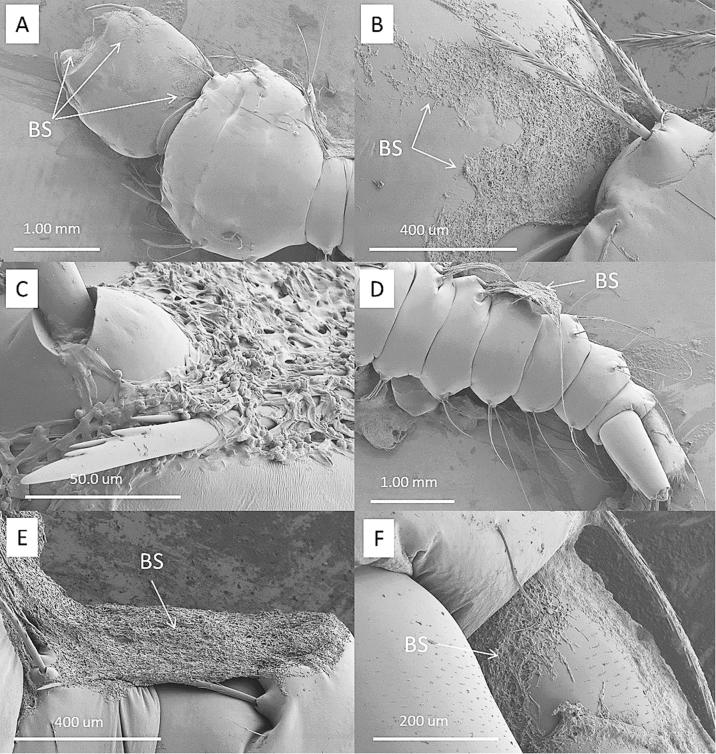
SEM of *Metarhizium brunneum* blastospores on *Toxorhynchites brevipalpis* larvae, 24 h post inoculation. Blastospores attached to mouthparts (A) head (A–B), abdomen setae (C–E) and siphon (F).

**Fig. 4:**
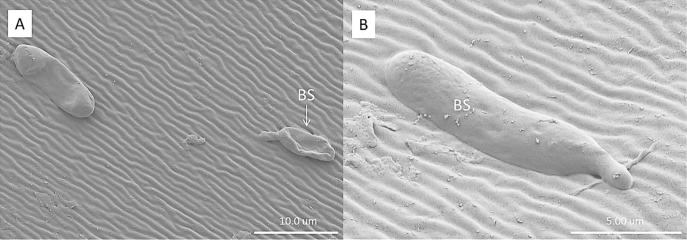
SEM of *Metarhizium brunneum* blastospores at the surface of the *Toxorhynchites brevipalpis* larval cuticle. Blastospores varied in size (A). Blastospores produced germ tubes which appear to be penetrating the host cuticle (A, B).

**Fig. 5:**
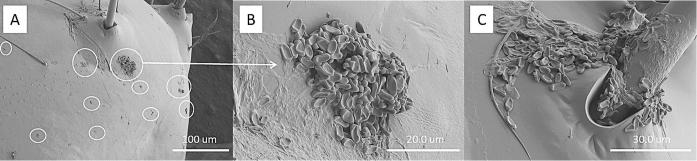
SEM of *Metarhizium brunneum* conidia on *Toxorhynchites brevipalpis* larvae, 24 h post inoculation, 24 h post inoculation. Conidia readily adhered to the cuticle surface either individually or in clusters (A). Close examination of the conidia showed that they had not germinated (B, C). Conidia often attached to or near the base of setae (C).

**Fig. 6:**
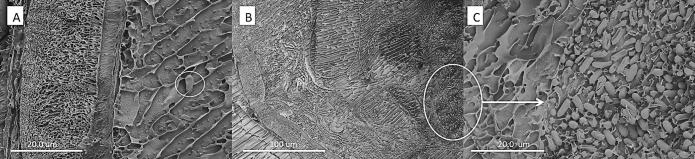
SEM of cross section of infected *Toxorhynchites brevipalpis* larvae with conidia of *Metarhizium brunneum.* (A) Conidia were present in very low quantities in the gut of the predator. (B–C) Large quantities of conidia were found in the gut of *A. aegypti* larvae that had been ingested by *Tx. brevipalpis* larvae.

Cryo-SEM showed that the hydrophobic conidia and hydrophilic blastospores of *M. brunneum* adhered to the surface of the cuticle of *T. brevipapis*. Blastospores adhered strongly to the head and mouthparts as well as abdominal setae and siphon (Fig. 3A–F). Blastospores were often observed in clumps with individual cells being connected by sheets or strands of mucilage (Fig. 3B–F). Isolated blastospores producing penetration hyphae were observed (Fig. 4A, B). Conidia of *M. brunneum* appeared to adhere through hydrophobic forces, often in clusters on or near the base of setae (Fig. 5A–C). There was no evidence of conidia germinating and producing germ tubes or appressoria beyond the first 24 h post-inoculation (pi). Conidia were clearly visible in the gut lumen of *T. brevipalpis* but none of these germinated or infected through the midgut epithelium (Fig. 6A–C).

**Fig. 7:**
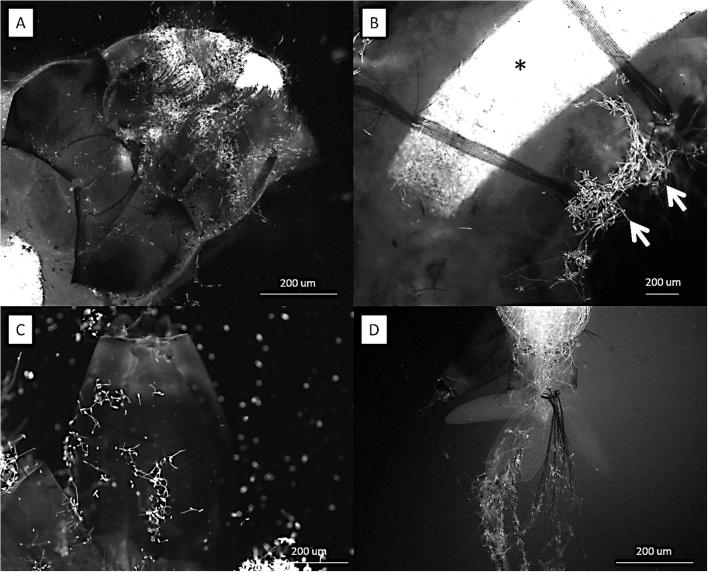
*Metarhizium brunneum* blastospores expressing GFP in the *Aedes aegypti* cuticle surface and the gut. Larvae inoculated with blastospores of a GFP transformed strain of *M. brunneum* were examined 24 h hr pi. Blastospores were attached to the head (A). They were visible at the surface of the abdomen (arrow) and in the gut (*) of ingested *Aedes* larvae (B). The blastospores also adhered to the surface of the siphon (C) and anal gills (D).

Blastospores adhered to the *A. aegypti* cuticle surface but were also concentrated in the gut lumen at 24 h pi. They would penetrate through the gut lumen and invade the haemocoel (Fig. 7A–D). In contrast, conidia of *M. brunneum* did not adhere to the cuticle surface of *A. aegypti* larvae but were ingested and concentrated in the gut lumen. They did not germinate in the gut lumen.

**Fig. 8:**
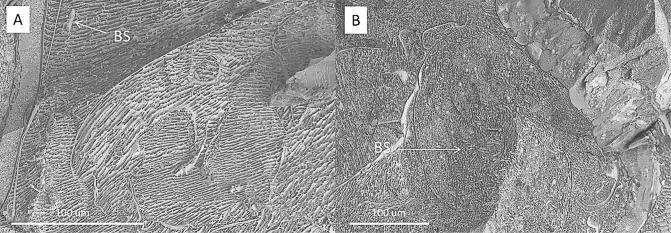
SEM of cross section of infected *Toxorhynchites brevipalpis* larvae with blastospores of *Metarhizium brunneum.* Very few blastopores were present in the gut of the predator (A). In contrast, a large number of blastospores were present in the gut of *A. aegypti *larvae, which had been ingested by *Tx. brevipalpis* (B).

**Fig. 9:**
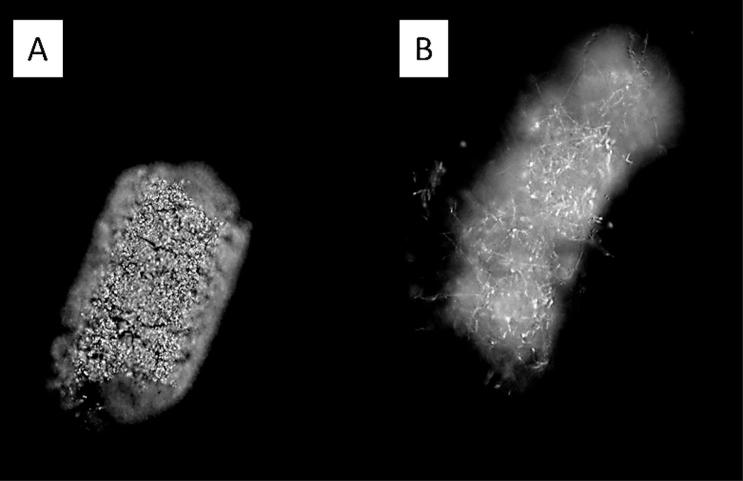
*Metarhizium brunneum* conidia and blastospores expressing GFP in faecal pellets of *Toxorhynchites brevipalpis*. *Tx. brevipalpis* Larvae were fed on *A. aegypti* larvae, which were inoculated with conidia and blastospores of a GFP transformed strain of *M. brunneum.* Faecal pellet being expelled from an infected larva showing many active conidia and blastospores.

Cross sections of the *T. brevipalpis* gut lumen showed ingested *A. aegypti* larvae at different stages of digestion. Recently ingested *A. aegypti* larvae had intact gut structure and content, with conidia or blastospores clearly visible in the gut lumen (Fig. 8, Fig. 6). Few spores were observed in the gut lumen of *T. brevipalpis* larvae; some may have been ingested while others were probably released from the prey during the digestive process. Fluorescence microscopy showed that both conidia and blastospores are expelled relatively intact in faecal pellets of *T. brevipalpis* larvae (Fig. 9A, B). Spores which expressed the GFP were clearly viable and active while the non-fluorescing GFP spores were probably quiescent or damaged and, therefore, non-viable (Fig. 9A and B).

### 3.3 Interaction between *M. Brunneum* and *T. Brevipalpis*

**Fig. 10:**
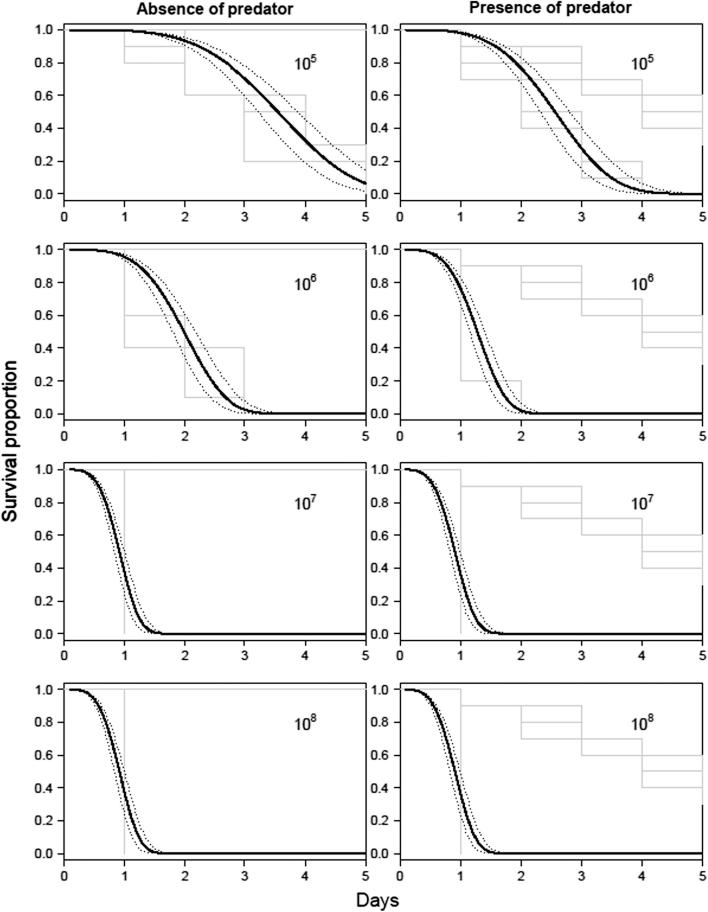
Survival curves of *Aedes aegypti* larvae exposed blastospores of *Metarhizium brunneum* with and without *Toxorhynchites brevipalpis*. Cumulative survival curves of *A. aegypti* treated with four different concentrations of *M. brunneum* (10^5^, 10^6^, 10^7^, 10^8^ blastospores ml^−1^) with one larvae of *Tx. brevipalpis* or without for five days. The negative control was distilled water. Fitted survival curves are shown in black, with 95% confidence intervals shown as dotted lines.

**Fig. 11:**
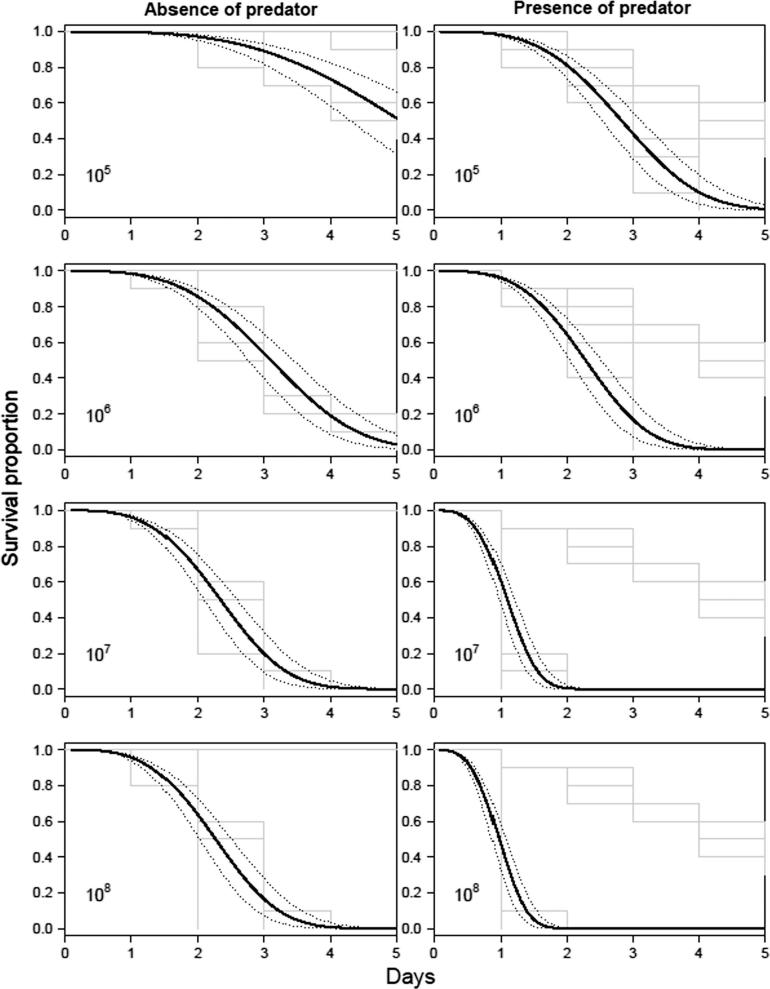
Survival curves of *Aedes aegypti* larvae exposed to conidia *Metarhizium brunneum* with and without *Toxorhynchites brevipalpis*. Cumulative survival curves of *A. aegypti* treated with four different concentrations of *M. brunneum* (10^5^, 10^6^, 10^7^, 10^8^ conidia ml^−1^) with one larvae of *Tx. brevipalpis* or without for five days. The negative control was distilled water. Fitted survival curves are shown in black, with 95% confidence intervals shown as dotted lines.

In the absence of *M. brunneum* ARSEF4556, all *A. aegypti* larvae survived 5 days incubation (Fig. 10, Fig. 11). However, when incubated with a single *T. brevipalpis* larva, ca. 67% were consumed (Fig. 4), with the differences between these controls being statistically significant (χ^2^ = 30.150, *df* = 3, *P* < .001; Table 4). Irrespective of fungal formulation (conidia or blastospores), survival of *A. aegypti* larvae was significantly lower when using combinations of *M. brunneum* and *T. brevipalpis* than with *T. brevipalpis* alone (Table 3, Table 4; Fig. 10, Fig. 11).

**Table 3:** Median lethal time (LT_50_) for *Aedes aegypti* larvae treated with blastospore and conidial formulations at 10^5^, 10^6^, 10^7^ and 10^8^ spores/ml in presence and absence of *Toxorhynchites brevipalpis* larvae.

Formulation	Concentration	LT_50_	
		Without *Tx. brevipalpis*	With *Tx. brevipalpis*
Blastospores	10^5^	3.89 (3.53–4.25)	2.82 (2.55–3.08)
	10^6^	2.17 (1.96–2.37)	1.41 (1.27–1.54)
	10^7^	1.00 (0.91–1.09)	1.00 (0.91–1.09)
	10^8^	1.00 (0.91–1.09)	1.00 (0.91–1.09)

Conidia	10^5^	5.64 (4.79–6.49)	3.15 (2.82–3.48)
	10^6^	3.45 (3.08–3.82)	2.54 (2.27–2.80)
	10^7^	2.60 (2.33–2.88)	1.22 (1.09–1.35)
	10^8^	2.52 (2.25–2.79)	1.09 (0.97–1.21)

Mean lethal time (LT_50_) for blastospores and conidial suspension with and without *Tx. brevipalpis* larvae versus *A. aegypti* larvae. 95% confidence intervals are given in parenthesis.

**Table 4:** Mortality rates (mean ± SEM) and Kaplan Meier Log-rank pairwise comparisons of *Aedes aegypti* larvae exposed to different concentrations of blastospores and conidia (1 × 10^5^, 1 × 10^6^, 1 × 10^7^, and 1 × 10^8^ ml^−1^) of *Metarhizium brunneum* for 5 days in the presence and absence of *Toxorhynchites brevipalpis* larvae.

Formulations		Concentrations			
Blastospores	10^5^		Control + T	10^5^	10^5^ + T
		Control	χ^2^ = 30.15 *P* < .001	χ^2^ = 63.86 *P* < .001	χ^2^ = 65.21 *P* < .001
		Control + T	–	χ^2^ = 8.78 *P* = .003	χ^2^ = 22.83 *P* < .001
		10^5^	–	–	χ^2^ = 8.72 *P* = .003
	10^6^		Control + T	10^6^	10^6^ + T
		Control	χ^2^ = 30.15 *P* < .001	χ^2^ = 65.25 *P* < .001	χ^2^ = 66.05 *P* < .001
		Control + T	–	χ^2^ = 33.75 *P* < .001	χ^2^ = 46.22 *P* < .001
		10^6^	–	–	χ^2^ = 9.90 *P* = .002
	10^7^		Control + T	10^7^	10^7^ + T
		Control	χ^2^ = 30.12 *P* < .001	χ^2^ = 59.00 *P* < .001	χ^2^ = 59.00 *P* < .001
		Control + T	–	χ^2^ = 48.27 *P* < .001	χ^2^ = 48.27 *P* < .001
		10^7^	–	–	NS
	10^8^		Control + T	10^8^	10^8^ + T
		Control	χ^2^ = 30.12 *P* < .001	χ^2^ = 59.00 *P* < .001	χ^2^ = 59.00 *P* < .001
		Control + T	–	χ^2^ = 48.27 *P* < .001	χ^2^ = 48.27 *P* < .001
		10^8^	–	–	NS

Conidia	10^5^		Control + T	10^5^	10^5^ + T
		Control	χ^2^ = 30.15 *P* < .001	χ^2^ = 19.80 *P* < .001	χ^2^ = 62.51 *P* < .001
		Control + T	–	NS	χ^2^ = 16.16 *P* < .001
		10^5^	–	–	χ^2^ = 31.46 *P* < .001
	10^6^		Control + T	10^6^	10^6^ + T
		Control	χ^2^ = 30.15 *P* < .001	χ^2^ = 65.77 *P* < .001	χ^2^ = 59.14 *P* < .001
		Control + T	–	χ^2^ = 12.14 *P* < .001	χ^2^ = 24.09 *P* < .001
		10^6^	–	–	χ^2^ = 6.49 *P* = .011
	10^7^		Control + T	10^7^	10^7^ + T
		Control	χ^2^ = 30.15 *P* < .001	χ^2^ = 62.79 *P* < .001	χ^2^ = 65.70 *P* < .001
		Control + T	–	χ^2^ = 23.48 *P* < .001	χ^2^ = 48.67 *P* < .001
		10^7^	–	–	χ^2^ = 42.15 *P* < .001
	10^8^		Control + T	10^8^	10^8^ + T
		Control	χ^2^ = 30.15 *P* < .001	χ^2^ = 63.14 *P* < .001	χ^2^ = 62.30 *P* < .001
		Control + T	–	χ^2^ = 24.37 *P* < .001	χ^2^ = 48.9 *P* < .001
		10^8^	–	–	χ^2^ = 44.61 *P* < .001

Statistical significance (*P* value) between *A. aegypti* larvae incubated with and without *Tx. brevipalpis* larvae (T) under infection with different concentration of *M. brunneum* conidia and blastospores. NS = not significant and χ^2^ = *Chi*-square value.

**Fig. 12:**
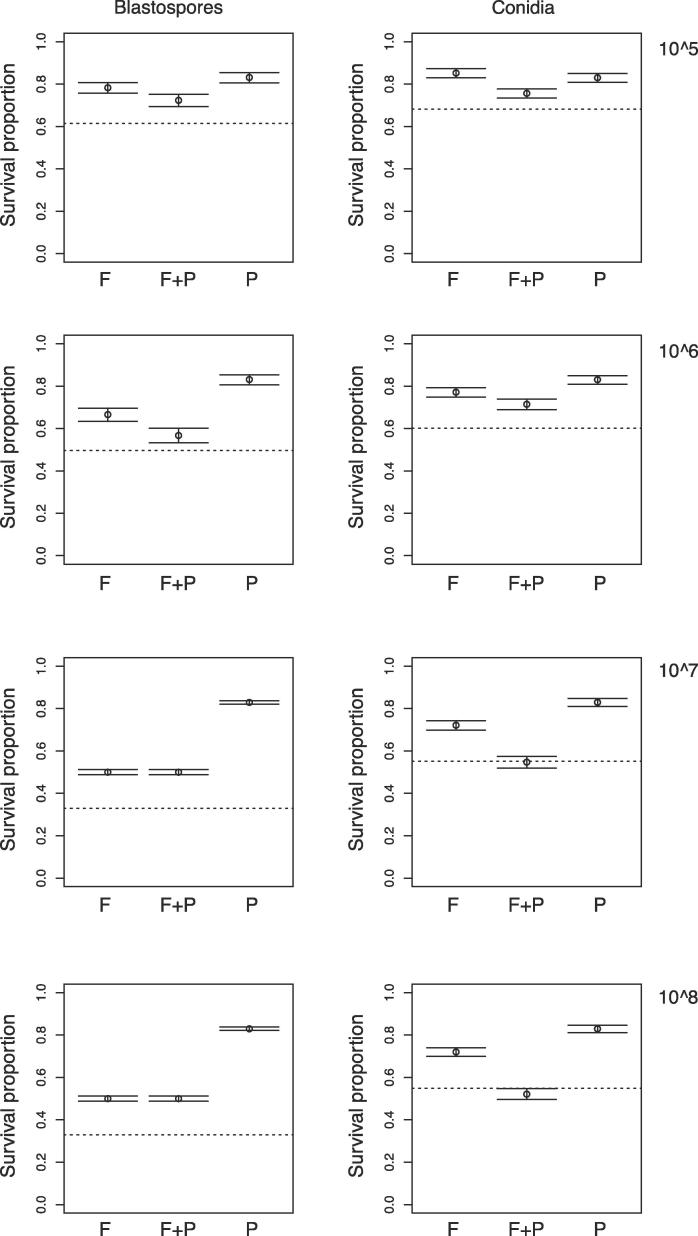
The interaction between *Metarhizium brunneum* treatments (blastospores – left-hand panels, and conidial – right-hand panels) and *Toxorhynchites brevipalpis* on survival of *Aedes aegypti* larvae*.* Survival proportion (mean with 95% confidence intervals) of *A. aegypti* treated with: 1) four concentrations of the fungus (“F”), *M. brunneum* (10^5^, 10^6^, 10^7^, 10^8^ spore ml^−1^), alone; 2) the fungus combined with one larva of the predator (“F + P”), *Tx. brevipalpis*; and 3) one larva of the predator (“P”), *Tx. brevipalpis* alone*.* The dotted line represents the expected level of the survival when the combination of fungus and predator are simply additive.

The interactions between these two biocontrol agents, as seen in Fig. 12, were antagonistic at the low concentrations (10^5^ and 10^6^ spores ml^−1^) for both the blastospore and conidia formulations. Antagonism increased with blastospore concentration (Fig. 12), where *A. aegypti* larvae survival was similar in the presence or absence of the predator at 10^7^ and 10^8^ spores ml^−1^ (Table 3). However, with conidial treatment, the combined effect of fungus and predator increased at higher fungal concentrations, to the point where the interaction was additive at 10^7^ spores ml^−1^ and synergistic at 10^8^ spores ml^−1^ (Fig. 12).

## 4 Discussion

Mycoinsecticides based on strains of EPF belonging to the genera *Metarhizium*, *Beauveria*, *Isaria* and *Lecanicillium* are either formulated as conidia or blastospores (de Faria and Wraight, 2007, Ravensberg, 2011). The latter is the preferred choice since it is comparatively cheaper e to produce and is generally more virulent (Alkhaibari et al., 2016, Behle et al., 2006). The current study shows that *M. brunneum* ARSEF 4556 blastospores are more virulent than the conidia against *T. brevipalpis* and *A. aegypti.* However, *T. brevipalpis* was significantly more tolerant than *A. aegypti* to both formulations at all the concentrations tested. However, when a combination of *M. brunneum* conidia or blastospores, used at low concentrations, and *T. brevipalpis* together resulted in significantly higher control of *A. aegypti* than using either agent alone.

Differences in pathogenesis could not entirely explain the differential susceptibility of these mosquito species. For example, conidia adhered to the surface of *T. brevipalpis* but not *A. aegypti*; this should have accelerated mortality of *T. brevipalpis* but no obvious infection structures (i.e. appressoria, penetrating hyphae) were observed questioning whether this was the route the fungus killed this predator. Presumably, conidia adhered but did not perceive the right cues to facilitate penetration of the cuticle (Butt et al., 2016). Conidia fail to adhere to the surface of *A. aegypti* due to weak adhesion forces (Greenfield et al., 2014). In contrast, the sticky, mucilage-producing blastospores firmly adhered to the surfaces of both mosquito species and appeared to have the capacity to penetrate the host cuticle and could account for the high mortality of this particular formulation (Alkhaibari et al., 2016).

Conidia and blastospores were readily ingested by *A. aegypti* but not in *T. brevipalpis*, reflecting differences in feeding mechanisms of these two species. The latter grabs and chews on its prey while *Aedes* species browse and filter food. Some propagules may enter the digestive tract when the predator starts to feed on mosquito prey but the majority of propagules are probably released during the digestion process. The fact that viable propagules were present in faecal pellets suggests that they are not digested.

The current study suggests that blastospores infect *T. brevipalpis* via the cuticle but not midgut epithelium. In contrast, blastospores can infect through both the cuticle and midgut epithelium of *A. aegypti* larvae, resulting in accelerated mortality (Alkhaibari et al., 2016). It is unclear if ingested conidia cause stress-induced mortality in *T. brevipalpis* as reported for *A. aegypti* larvae (Butt et al., 2013). In the latter case, conidia do not germinate in the gut lumen but the spore bound protease, Pr1, triggers stress induced apoptosis ultimately leading to death (Butt et al., 2013). The fact that *T. brevipalpis* mortality increased with concentration suggests that the conidia may have contributed to the mortality via this mechanism albeit with the conidia mostly being derived from the prey during the digestion process.

This study shows that the potential exists for the combined use of *M. brunneum* ARSEF 4556 and *T. brevipalpis* to control *A. aegypti* larvae. Combinations of these two biocontrol agents can potentially be antagonistic (weaker than additive), additive, or synergistic (stronger than additive) (Koppenhöfer and Kaya, 1997). The current study shows that significant reductions in lethal times were achieved by combining *M. brunneum* conidia with *T. brevipalpis* over a wide range of fungal concentrations, compared to fungal treatment alone. While beneficial, this interaction proved to be antagonistic at lower fungal conidia concentrations, but becoming at least additive at higher concentrations. However, when blastospores were used, addition of *T. brevipalpis* was only advantageous (but antagonistic) over fungus treatment alone at lower fungal concentrations, with no additional effects of the predator over fungus alone at the highest concentrations. The increasing antagonism between predator and blastospores may have been simply due to the fast action of the fungus in killing *A. aegypti* larvae before the predators had any additional effect, or due the fungus directly affecting the predators. In contrast, the combined effects of the conidia and predator were stronger with increasing fungal dosage. Many interacting factors can influence the combined effects of fungus and predator. For example, if the predator bites but does not kill its larval prey, then the fungus may find a way in through the wound and accelerate death (Wu et al., 2015). However, injury will activate phenoloxidase leading to production of melanin and precursors which are toxic to fungi (Tanada and Kaya, 2012, Butt et al., 2016). Furthermore, fungal infection may reduce larval mobility, so increasing their susceptibility to predation (Gehman and Byers, 2017).

Clearly the potential exists to develop IVM strategies targeting mosquito larvae through careful selection of the optimal concentration and formulation of *M. brunneum*. The laboratory findings may not always reflect what happens in the field due to a range of environmental factors. However, they do illustrate the sort of scenarios that likely take place in the field. Thus the fungus could be applied alone at low concentrations to work in concert with natural populations of *Toxorhynchites* with little risk to the latter. Alternatively, synergy between *M. brunneum* and *Toxorhynchites* could be exploited by using low concentrations of the fungus with concomitant introduction of the predator. The approaches outlined above will reduce costs, accelerate control, and concomitantly reduce risks to beneficial mosquito predators such as *Toxorhynchites*. Indeed, reduced application rates have been shown to reduce risks to several aquatic non-target aquatic invertebrates (Garrido-Jurado et al., 2015). In urban areas where rapid “knockdown” of a mosquito population is often necessary then high concentrations of *M. brunneum* blastospores would be required. However, there are many other situations where regular application of EPF would be required, for example: to prevent mosquito establishment, eradication of invasive species or suppression of mosquito populations (cryptic habitats, remote rural habitats) to pre-empt sudden outbreaks following rainfall or flooding. IVM programmes could be improved through a thorough understanding of interactions between EPF and mosquito predators whether natural or introduced.

## References

[b0005] Alkhaibari A., Carolino A., Bull J., Samuels R., Butt T. (2017). Differential pathogenicity of *Metarhizium* blastospores and conidia against larvae of three mosquito species. J. Med. Entomol..

[b0010] Alkhaibari A.M., Carolino A.T., Yavasoglu S.I., Maffeis T., Mattoso T.C., Bull J.C., Samuels R.I., Butt T.M. (2016). *Metarhizium brunneum* blastospore pathogenesis in *Aedes aegypti* Larvae: attack on several fronts accelerates mortality. PLoS Pathog..

[b0015] Ansari M., Shah F., Butt T. (2008). Combined use of entomopathogenic nematodes and *Metarhizium anisopliae* as a new approach for black vine weevil, *Otiorhynchus sulcatus*, control. Entomol. Exp. Appl..

[b0020] Becker N., Petrić D., Boase C., Lane J., Zgomba M., Dahl C., Kaiser A. (2003).

[b0025] Behle R., Garcia-Gutierrez C., Tamez-Guerra P., McGuire M., Jackson M. (2006). Pathogenicity of blastospores and conidia of *Paecilomyces fumosoroseus* against larvae of the Mexican bean beetle, *Epilachna varivestis* Mulsant. Southwestern Entomol..

[b0030] Boyer S., Paris M., Jego S., Lempérière G., Ravanel P. (2012). Influence of insecticide *Bacillus thuringiensis subsp. israelensis* treatments on resistance and enzyme activities in *Aedes rusticus* larvae (Diptera: Culicidae). Biol. Control..

[b0035] Butt T., Coates C., Dubovskiy I., Ratcliffe N. (2016). Chapter nine-entomopathogenic fungi: new insights into host-pathogen interactions. Adv. Genet..

[b0040] Butt T.M., Greenfield B.P., Greig C., Maffeis T.G., Taylor J.W., Piasecka J., Dudley E., Abdulla A., Dubovskiy I.M., Garrido-Jurado I. (2013). *Metarhizium anisopliae* pathogenesis of mosquito larvae: a verdict of accidental death. PLoS ONE.

[b0045] Ceretti-Junior W., de Oliveira Christe R., Rizzo M., Strobel R.C., de Matos Junior M.O., de Mello M.H.S.H., Fernandes A., Medeiros-Sousa A.R., de Carvalho G.C., Marrelli M.T. (2016). Species composition and ecological aspects of immature mosquitoes (Diptera: Culicidae) in bromeliads in urban parks in the city of Sao Paulo, Brazil. J. Arthropod. Borne Dis..

[b0050] Collins L.E., Blackwell A. (2000). The biology of *Toxorhynchites* mosquitoes and their potential as biocontrol agents. Biocontrol News Inform..

[b0055] Crawley M. (2012). The R Book, Edn.

[b0060] de Faria M.R., Wraight S.P. (2007). Mycoinsecticides and mycoacaricides: a comprehensive list with worldwide coverage and international classification of formulation types. Biol. Control..

[b0065] Dogan, Y., Hazir, S., Yildiz, A., Butt, T.M., Cakmak, I., 2017. Evaluation of entomopathogenic fungi for the control of *Tetranychus urticae* (Acari: Tetranychidae) and the effect of *Metarhizium brunneum* on the predatory mites (Acari: Phytoseiidae). Biol. Control.

[b0070] Faull K., Webb C., Williams C. (2016). Desiccation survival time for eggs of a widespread and invasive Australian mosquito species, *Aedes* (Finlaya) *notoscriptus* (Skuse). J. Vector Ecol..

[b0075] Fillinger U., Ndenga B., Githeko A., Lindsay S.W. (2009). Integrated malaria vector control with microbial larvicides and insecticide-treated nets in western Kenya: a controlled trial. Bull. World Health Organ..

[b0080] Garrido-Jurado I., Alkhaibari A., Williams S., Oatley-Radcliffe D., Quesada-Moraga E., Butt T. (2015). Toxicity testing of *Metarhizium* conidia and toxins against aquatic invertebrates. J. Pest. Sci..

[b0085] Gehman A.-L.M., Byers J.E. (2017). Non-native parasite enhances susceptibility of host to native predators. Oecol..

[b0090] Greenfield B.P., Lord A.M., Dudley E., Butt T.M. (2014). Conidia of the insect pathogenic fungus, *Metarhizium anisopliae*, fail to adhere to mosquito larval cuticle. R. Soc. Open Sc.i.

[b0095] Greenfield B.P., Peace A., Evans H., Dudley E., Ansari M.A., Butt T.M. (2015). Identification of *Metarhizium* strains highly efficacious against *Aedes, Anopheles* and *Culex* larvae. Biocontrol Sci. Technol..

[b0100] Hemingway J., Ranson H. (2000). Insecticide resistance in insect vectors of human disease. Annu. Rev. Entomol..

[b0105] Ippolito A., Kattwinkel M., Rasmussen J.J., Schäfer R.B., Fornaroli R., Liess M. (2015). Modeling global distribution of agricultural insecticides in surface waters. Environ. Pollut..

[b0110] Juliano S.A., O'Meara G.F., Morrill J.R., Cutwa M.M. (2002). Desiccation and thermal tolerance of eggs and the coexistence of competing mosquitoes. Oecology.

[b0115] Kaplan E.L., Meier P. (1958). Nonparametric estimation from incomplete observations. J. Am. Stat. Assoc..

[b0120] Koppenhöfer A., Kaya H. (1997). Additive and Synergistic Interaction between Entomopathogenic Nematodes and *Bacillus thuringiensis* for Scarab Grub Control. Biol. Control..

[b0125] Lacey L.A. (2007). *Bacillus thuringiensis* serovariety israelensis and *Bacillus sphaericus* for mosquito control. J. Am. Mosq. Control Assoc..

[b0130] Liess M., Foit K., Becker A., Hassold E., Dolciotti I., Kattwinkel M., Duquesne S. (2013). Culmination of low-dose pesticide effects. Environ. Sci. Technol..

[b0135] Meyling N.V., Pell J.K. (2006). Detection and avoidance of an entomopathogenic fungus by a generalist insect predator. Ecol. Entomol..

[b0140] Mohamad N., Zuharah W. (2014). Influence of container design on predation rate of potential biocontrol agent, *Toxorhynchites splendens* (Diptera: Culicidae) against dengue vector. Trop. biomed..

[b0145] Morgan G.A., Leech N.L., Gloeckner G.W., Barrett K.C. (2012). IBM SPSS for Introductory Statistics: Use and Interpretation.

[b0150] Mulla M.S., de Barjac H., Sutherland D.J. (1990). Activity, field efficacy, and use of *Bacillus thuringiensis israelensis* against mosquitoes. Bacterial Control of Mosquitoes & Black Flies.

[b0155] Nkya T.E., Poupardin R., Laporte F., Akhouayri I., Mosha F., Magesa S., Kisinza W., David J.P. (2014). Impact of agriculture on the selection of insecticide resistance in the malaria vector Anopheles gambiae: a multigenerational study in controlled conditions. Parasit. Vectors..

[b0160] Ormond E.L., Thomas A.P., Pell J.K., Freeman S.N., Roy H.E. (2011). Avoidance of a generalist entomopathogenic fungus by the ladybird, *Coccinella septempunctata*. FEMS. Microbiol Ecol..

[b0165] R Core Team, 2012. R: A language and environment for statistical computing. R Foundation for Statistical Computing, Vienna, Austria. ISBN 3-900051-07-0, URL http://www.R-project.org.

[b0170] Ravensberg W.J. (2011). A Roadmap to the Successful Development and Commercialization of Microbial Pest Control Products for Control of Arthropods.

[b0175] Roy H.E., Pell J.K. (2000). Interactions between entomopathogenic fungi and other natural enemies: implications for biological control. Biocontrol Sci. Technol..

[b0180] Saito T., Brownbridge M. (2016). Compatibility of soil-dwelling predators and microbial agents and their efficacy in controlling soil-dwelling stages of western flower thrips *Frankliniella occidentalis*. Biol. Control..

[b0185] Scholte E.-J., Knols B.G., Samson R.A., Takken W. (2004). Entomopathogenic fungi for mosquito control: a review. Insect Sci.

[b0190] Shaalan E.A.-S., Canyon D.V. (2009). Aquatic insect predators and mosquito control. Trop Biomed.

[b0195] Shah P., Pell J. (2003). Entomopathogenic fungi as biological control agents. Appl. Microbiol. Biotechnol..

[b0200] Tanada Y., Kaya H.K. (2012). Insect Pathology.

[b0205] Thomas M.B. (2017). Biological control of human disease vectors: a perspective on challenges and opportunities. Biocontrol.

[b0210] Tolle M.A. (2009). Mosquito-borne diseases. Curr. Probl. Pediatr. Adolesc. Health Care.

[b0215] Wu, S.-y., Y.-l. GAO, X.-n. XU, M.S. Goettel, and Z.-r. LEI. 2015. Compatibility of *Beauveria bassiana* with *Neoseiulus barkeri* for control of *Frankliniella occidentalis. J. Integr. Agric.* 14:98-105.

